# The 2024 Arnold Berliner award on solitary bee behavior and a farewell

**DOI:** 10.1007/s00114-024-01940-6

**Published:** 2024-09-27

**Authors:** Matthias Waltert

**Affiliations:** Department of Conservation Biology, University of Göttingen, Bürgerstrasse 50, 37073 Göttingen, Germany

**Fig. 1:**
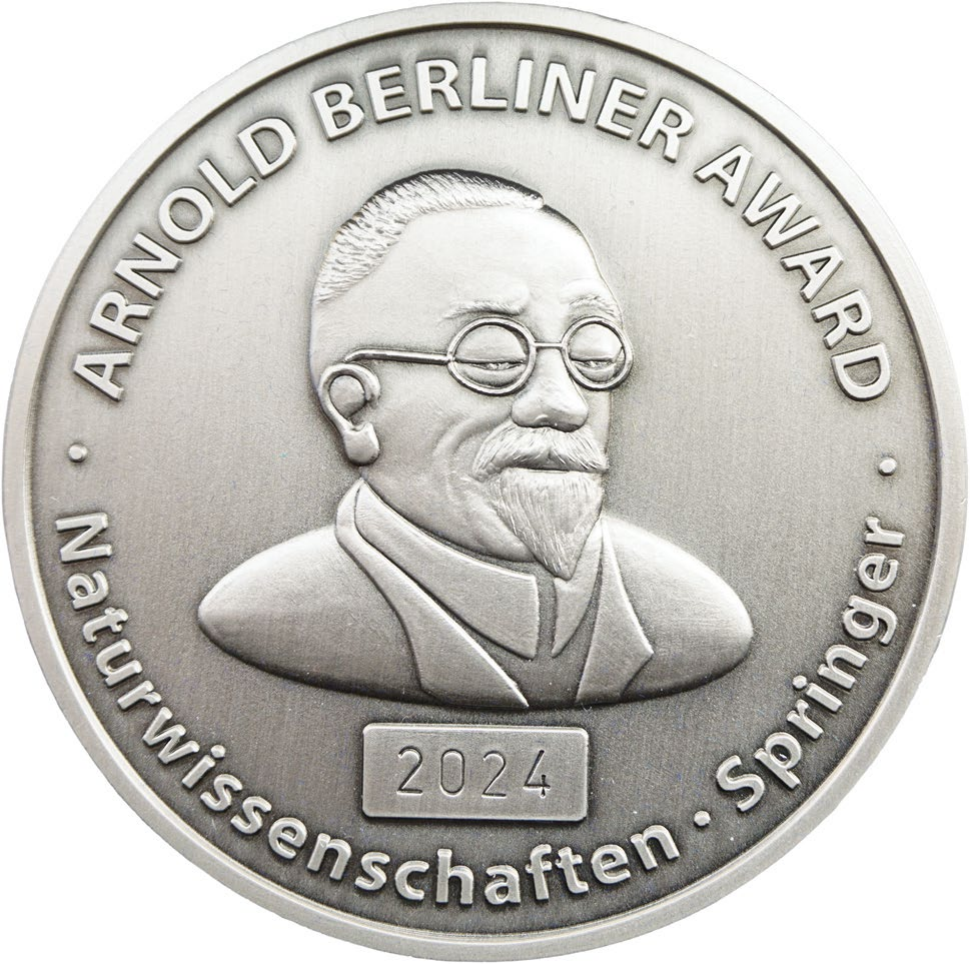
Arnold Berliner Award medal

As it has for several years, *The Science of Nature* grants the Arnold Berliner Award to the lead author of an article which represents excellent, original, and—especially—interdisciplinary research. As such, each winning article clearly reflects the vision of the founder of our journal, Arnold Berliner (Autrum 1988; Thatje 2018). Springer sponsors the award which includes four parts: the Arnold Berliner Award medal (Fig. 1), a 2-year subscription to the journal’s online edition, a 500-Euro voucher for Springer ebooks, and a cash prize of 250 Euro.

**Fig. 2:**
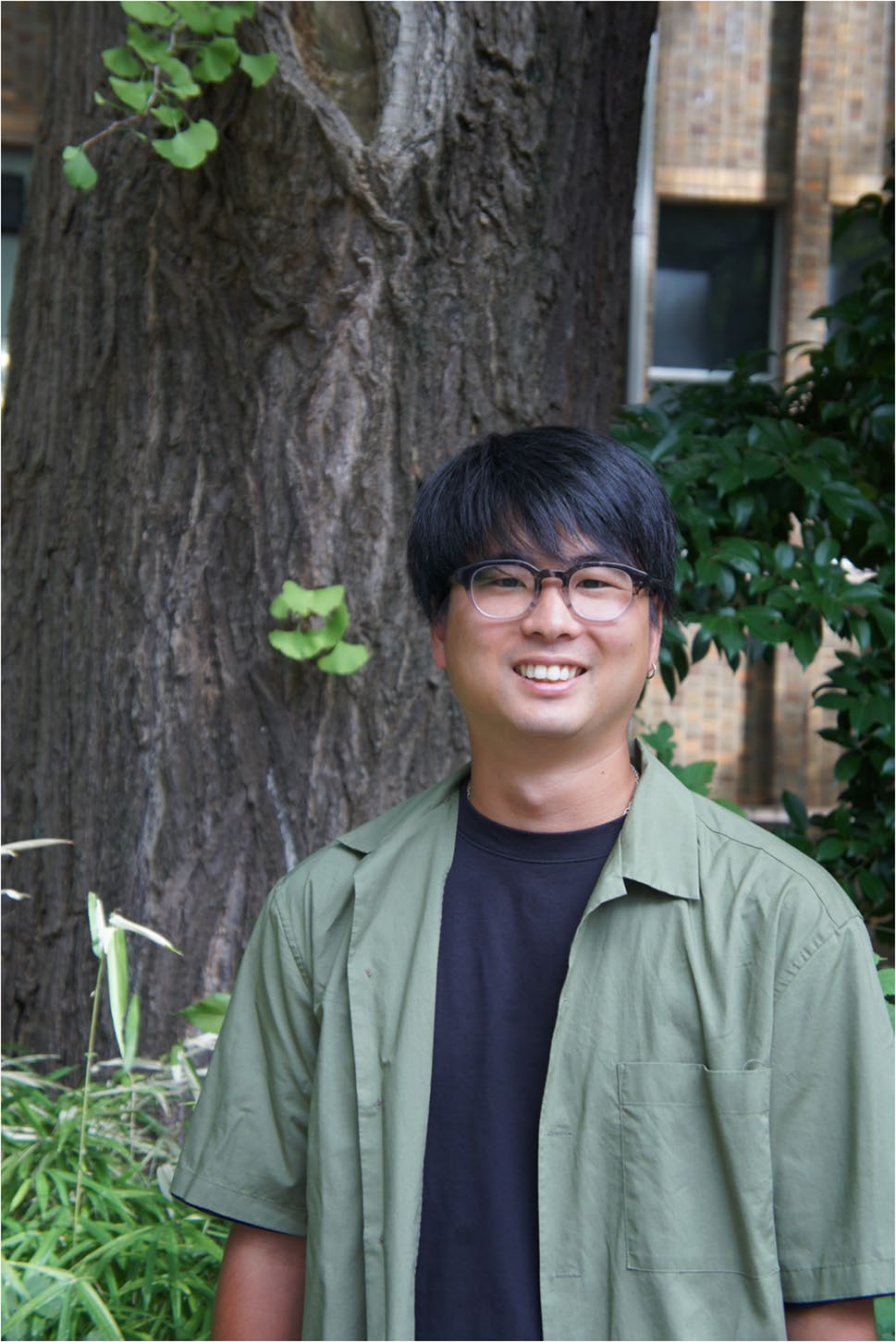
Yuta Nagano

I am very proud that this year, the editorial board of our journal has decided to award Yuta Nagano (Fig. 2) for his article “Female solitary bees flexibly change foraging behaviour according to their floral resource requirements and foraging experiences” (Nagano et al. 2023). With their research, the authors show that solitary bees are able to adjust their foraging behavior according to their individual experience and nutritional needs. The research integrates a variety of datasets such as on behavior, morphological changes associated with foraging experience, and analysis of the gut contents. This study is an exceptional example for the value of research on understudied taxa: solitary bees provide important ecosystem services at the global level but have not received sufficient research attention, most probably also because of their often wrongly perceived economic importance. On behalf of the editorial board, I congratulate Yuta Nagano, as well as his coauthors Naoto Wabiko and Tomoyoki Yokoi, for this article and on the award.

This editorial is also a farewell. I am filled with a profound sense of gratitude to have been able to lead this exceptional journal: I will end my role as editor in chief at the end of this year. It has been an extraordinary privilege to serve *The Science of Nature* for two 3-year terms. During this time, I have witnessed the journal grow, evolve, and make significant strides in the realm of scientific publishing. As I now prepare to pass the torch to my successor, I would like to reflect on our journey together and express my heartfelt thanks to the incredible community that has supported us.

When I assumed this role, my vision for *The Science of Nature* was to foster a periodical that not only disseminates biologically interesting phenomena but also helps the process of scientific publishing in a way that is equally fair to all involved actors. I am grateful to have been able to achieve this together with our authors, editors, publishing managers, and the production team. As an inclusive and collaborative scientific community, over the years, we together have maintained a broad horizon, embraced interdisciplinary thinking, and admired the organismic world. We have been driven by passion, dedication, and ingenuity of the researchers, reviewers, and editorial team who have worked tirelessly to uphold the journal’s standards of excellence.

One of the greatest joys of my tenure has been to oversee the broadness in the scope of the biological studies we published, always including research on organisms beyond those which are already established scientific models, and the ecological, evolutionary processes shaping their traits and behaviors. When Arnold Berliner established the journal, his intention was to communicate what is interesting in the natural world, and our authors have advanced such knowledge and inspired further inquiry. It is humbling to see how diverse the natural world is and how much knowledge can be sometimes gained from simple observations, which often lead to experimental approaches and a higher level of understanding of the mechanisms behind observed patterns.

*The Science of Nature’s* success is, without a doubt, a collective achievement. I am deeply grateful to our editorial board members, who have provided invaluable guidance and expertise. Their commitment to rigorous peer review and mentorship has ensured the integrity and quality of our publications. I also extend my sincere appreciation to our reviewers, whose thorough and constructive feedback has been instrumental in refining and enhancing the research we publish.

To our readers and authors, thank you for your unwavering trust and engagement. Your contributions and support have been the lifeblood of this journal. It has been an honor to provide a platform for your discoveries and to witness the vibrant exchange of ideas that define our scientific community.

As I step down from my role, I am confident that *The Science of Nature* will continue to thrive under new leadership. The incoming editor in chief brings a wealth of experience, fresh perspectives, and a shared commitment to advancing scientific knowledge. I have no doubt that the journal will reach even greater heights in the years to come.

In closing, I would like to emphasize again that the journey of discovery is a collective endeavor. The pursuit of knowledge is never-ending, and each of us plays a vital role in pushing the boundaries of what we know. As I bid farewell, I do so with immense pride in what we have accomplished together and with optimism for the future of *The Science of Nature*.

Thank you for allowing me the privilege to serve this remarkable community.

With deepest gratitude,

Matthias Waltert

Editor in Chief

*The Science of Nature*
